# Comparisons of Health Promoting Behavior, Depression, and Life Satisfaction Between Older Adults in Rural Areas in South Korea Living in Group Homes and at Home

**DOI:** 10.1097/JNR.0000000000000290

**Published:** 2019-05-20

**Authors:** Sohyune R. SOK, Bo Kyeong CHEON, Min Kyung GU, Ok Sun KIM

**Affiliations:** 1 PhD, RN, Professor, College of Nursing Science, Kyung Hee University, Seoul, Republic of Korea;; 2 MSN, RN, Doctoral Student, Department of Nursing, Graduate School, Kyung Hee University, Seoul, Republic of Korea;; 3 PhD, RN, Assistant Professor, Department of Nursing, Kyungbuk College, Gyeongbuk, Republic of Korea.

**Keywords:** aged, group homes, health promoting behavior, depression, life satisfaction

## Abstract

**Background:**

In South Korea, population aging is advancing at a more rapid rate in rural areas than urban areas, leading to a particularly high percentage of rural-dwelling older adults.

**Purpose:**

The aim of this study was to examine and compare health promoting behaviors, depression, and life satisfaction between rural-dwelling older adults who live, respectively, in group homes and at home.

**Methods:**

A cross-sectional descriptive study design was employed. Study participants included 160 older adults aged 65 years and older who were living in group homes (*n* = 80) and at home (*n* = 80) in Gyeonggi province, South Korea. The Health Promotion Lifestyle Profile-II was used to examine health promoting behaviors, the Korean Geriatric Depression Screening Scale was used to examine depression, and the Life Satisfaction Index was used to examine life satisfaction. Data were analyzed using SPSS Version 21.0.

**Results:**

The data showed significant differences between the two groups in terms of health promoting behaviors (*t* = −9.035, *p* < .001), depression (*t* = 20.861, *p* < .001), and life satisfaction (*t* = −12.153, *p* < .001).

**Conclusions/Implications for Practice:**

The mean scores for health promotion behaviors and life satisfaction were higher, and the mean score for depression was lower in the group-home group than the at-home group. The findings from this study may be employed as basic data for establishing residence-appropriate nursing intervention protocols for older adults living in rural areas.

## Introduction

The average life expectancy of South Koreans was 82.4 years old, and the older adult population (≥ 65 years old) accounted for 13.7% of the total population in 2016 (Korea National Statistical Office, 2016). In 2026, the number of older adults in the country is expected to reach about 20%, which will make South Korea a super-aged society (Korea National Statistical Office, 2016). In South Korea, the development of commerce and industry has caused a long-term and massive shift in population from rural areas to cities (Ko, 2016). Consequently, population aging has had a disproportional impact on rural areas (Jang & Kim, 2014), leaving rural South Korea with a relatively high percentage of older adults (Ko, 2016; Sok & Kim, 2008). Although public programs for the older adults exist, they are insufficient and narrow in scope (Jang & Kim, 2014).

Current South Korean housing may be roughly divided into two types: “silver towns,” which are expensive senior residential facilities, and free nursing homes, which are populated primarily by older adults from low-income families (E. H. Kim & Lee, 2009). Both types of facilities foster loneliness among their residents due to the necessity of living among strangers, fear due to isolation from family, and the burden of having to adapt to a new environment (Custers, Westerhof, Kuin, & Riksen-Walraven, 2010; H. S. Kim, 2016). Thus, they offer no benefit for most middle-class older adults (S. H. Lee, 2010). Admission to a facility means having to adapt to a new environment and leaving familiar dwellings (Heliker & Scholler-Jaquish, 2006; H. S. Kim, 2016), which may affect older adults psychologically and mentally by cutting off all of their interpersonal relationships (Custers et al., 2010; Van Zadelhoff, Verbeek, Widdershoven, van Rossum, & Abma, 2011) as well as negatively affect their physical status (H. S. Kim, 2016; Vogelsang, 2016). Therefore, some scholars have argued that older adults should continue to reside in living environments with which they are familiar and comfortable (Oh, 2008; Van Zadelhoff et al., 2011; Verbeek, Zwakhalen, van Rossum, Kempen, & Hamers, 2012). Thus, spaces are urgently needed where older adults may live comfortably in a home-like atmosphere (S. H. Lee, 2010; Sok & Yun, 2011; Te Boekhorst, Depla, Pot, de Lange, & Eefsting, 2011).

In South Korea, region-friendly, small-scale older adult group homes were established after the government enacted the Long-Term Care Insurance Act in July 2008 (Ministry of Health and Welfare, 2015). In most nursing homes for older adults, more than 10 senior citizens live as a group. By contrast, the post-July 2008 older adult group homes are designed to house small groups of around 10 persons, who live freely in a home-like atmosphere that is located in their original area of residence (Oh, 2008; Verbeek et al., 2012). Moreover, residents may pursue their own life interests, and family members and friends may come and go freely. These new older adult group homes target middle-class older adults, whereas the earlier nursing homes target low-income older adults (Oh, 2008; Te Boekhorst et al., 2011; Van Zadelhoff et al., 2011). After the enactment of the Long-Term Care Insurance Act in 2008, the number of older adults admitted to older adult group homes has gradually increased, from 618 in 2009 to 1,173 in 2014. The number of group home facilities has also increased from 75 in 2009 to 142 in 2014 (Ministry of Health and Welfare, 2015). Other countries have small-group-home programs similar to the South Korean program. After Sweden first introduced the group homes program in 1970, similar programs were adopted in other Nordic countries. Group homes were established as special nursing homes in the Netherlands in the 1980s and in Japan in 2002 (Te Boekhorst et al., 2011; Van Zadelhoff et al., 2011; Verbeek et al., 2012). Such group homes have advantages in that older adult residents may continue to use their existing community networks, which provide emotional stability (S. H. Lee, 2010; Te Boekhorst et al., 2011; Van Zadelhoff et al., 2011; Verbeek et al., 2012).

As noted above, South Korea’s older adult population is growing faster in terms of percentage in rural areas than urban areas. “Economic problems” is the greatest difficulty reported by urban older adults, whereas “heath problems” is the greatest difficulty reported by rural older adults (Korea National Statistical Office, 2016). Urban and rural areas present different living environments, and their residents have different lifestyles (Ko, 2016). Furthermore, the economic status of rural residents is generally lower than that of urban residents (Jang & Kim, 2014). Therefore, the health management abilities and health promotion behaviors of rural older adults invariably lag behind those of their urban-dwelling peers (Jang & Kim, 2014; Ko, 2016). Incorrect health promotion behaviors and lifestyles lead to chronic diseases (E. H. Kim & Lee, 2009). Poor health status reduces the physical activity of older adults, increasing perceived solitude and loneliness and dependence on family and decreasing life satisfaction in both the physical and functional dimensions (Custers et al., 2010; Hosseinpoor et al., 2016; Sok & Kim, 2008).

Most studies addressing this topic have compared older adults living in facilities such as nursing homes and silver towns with older adults living at home. Few studies have addressed older adults living in the abovementioned new group homes. However, the group homes program for older adults is a rising trend in rural areas (S. H. Lee, 2010; Oh, 2008; Van Zadelhoff et al., 2011). Thus, further studies on the health promotion behaviors, depression status, and life satisfaction of older adults living in these region-friendly, small, co-residential group homes are required, and such older adults need to be compared with those living at home in the rural areas.

The purpose of this study was to examine and compare the health promoting behavior, depression, and life satisfaction of older adults in rural areas in South Korea who, respectively, live in group homes and at home.

## Methods

### Participants

A cross-sectional descriptive study design was employed. Participants included 160 older adults aged 65 years and older who were living either in group homes (*n* = 80) or at home (*n* = 80) in Gyeonggi province, South Korea. The participants were recruited using convenience sampling. Eligibility criteria included being at least 65 years old, understanding the purpose of this study, providing informed consent to participate, having no cognitive impairment (a score of ≥ 24 on the Mini-Mental Status Examination-Korea), and having complete verbal communication ability in Korean. One hundred seventy questionnaires were issued, and 163 (95.88%; group homes: 96.47%, home: 95.29%) were returned, with 160 accepted as valid and included in the final data set and three rejected because of incompleteness.

Sample size adequacy (*n* = 64 in each group) was assed using an *F* test, and G*Power 3.1 analysis software was used, based on an alpha level = .05, a medium effect size = 0.3, and power = 0.8 (Faul, Erdfelder, Lang, & Buchner, 2007). The sample size in the study was determined to be adequate.

### Measures

The questionnaire was designed to measure general characteristics, health promoting behaviors, depression, and life satisfaction. General characteristics consisted of gender, age, education, religion, spouse, children, pocket money, number of persons living together, motivation for admission, admission period, and perceived health status. It consisted of 11 items.

Health Promotion Lifestyle Profile-II was developed by Walker, Sechrist, and Pender (1995) and revised by S. M. Lee (2012). The scale was used to measure the health promoting behavior of the participants and consisted of three subcategories (physical area, social area, and emotional area) and 13 questions that were scored using a 5-point Likert scale. The possible score range was 13–65, with higher scores associated with higher levels of health promoting behavior. The reliability of this instrument was Cronbach’s α = .91. The reliability of each subcategory was Cronbach’s α = .89, .82, and .86, respectively.

The Korean Geriatric Depression Screening Scale, developed by Jung et al. (1997), was used to measure level of depression and employed 30 questions answered by yes (1) or no (2). The possible score range was 30–60, and higher scores were associated with a higher level of depression. Scores of 28–37 were interpreted as borderline depression, scores of 38–43 were interpreted as moderate depression, and scores of 44 and higher were interpreted as severe depression. Instrument reliability was Cronbach’s α = .88.

The Life Satisfaction Index, developed by Neugarten, Havighurst, and Tobin (1961), was used to measure level of life satisfaction. This scale consisted of 14 questions that were scored using a 5-point Likert scale with a total possible score range of 14–70 and higher scores associated with better life satisfaction. The reliability of this instrument was Cronbach’s α = .92.

### Data Collection

The data collection period for this study was September to December 2015. The researcher visited two group homes for older adults in Gyeonggi province to obtain permission to conduct this study. There were no significant within-group-home differences in terms of basic demographic characteristics or location. After obtaining permission from the group homes, the researcher contacted the older adults living in these facilities. To survey older adults living at home, the researcher visited every house to invite qualified older adults to participate. The researcher contacted all of the prospective older adult participants living either in group homes or at home to explain the purpose and objective of the research, the details of participation, and the instruments that would be used. The participants were selected after receiving written informed consent. The questionnaire was given only to those older adults who agreed to participate, and the completed questionnaires were collected. The questionnaires were self-reported and administered by the researcher. Each participant took approximately 30–35 minutes to finish the survey.

### Data Analysis

Data were analyzed using the SPSS Version 21.0 (IBM, Armonk, NY, USA). Descriptive statistics, chi-squared test, independent *t* test, and Fisher exact test were used to analyze the demographic characteristics of the participants. To compare between the two groups, independent *t* test, *F* test, and analysis of covariance (ANCOVA) were used. A Scheffe test was used for the post hoc test, and ANCOVA statistics were used to control the baseline differences between people living in group homes and at home. The level of statistical significance was set at .05.

### Ethical Considerations

This study was approved by the institutional review board of a university in Seoul, South Korea (Approval no. KHSIRB-15-027). Participants were informed that they could voluntarily take part in this study and that they could also withdraw from participation at any time. Moreover, they were informed about the anonymity and the confidentiality of the data they would provide. The researchers obtained completed, written consent forms from the eligible subjects before their participation.

## Results

### General Characteristics of Study Participants

In terms of gender, 60.0% of the older adults living at home were female and 40.0% were male, whereas 65.0% of the older adults living in group homes were female and 35.0% were male. In terms of ages, the total mean age was 79.51 ± 4.32 years, the mean age of those living at home was 79.94 ± 4.46 years, and the mean age of those living in group homes was 79.08 ± 4.17 years. Nearly two fifths (38.8%) of the elderly people living at home had a spouse (61.2% did not), whereas only 13.8% of the older adults living in group homes had a spouse (86.2% did not). In terms of perceived health status, 59.9% of the at-home group indicated that they were not healthy and 40.1% indicated that they were, whereas 55.0% of the group-home group indicated that they were healthy and 45.0% indicated that they were not. Thus, more in the group-home group self-perceived as healthy. No significant differences were identified between the groups in terms of general characteristics, with the exception of having a spouse, living with another person, and perceived health status. Presence of spouse, living with another person, and perceived health status were controlled using ANCOVA (Table 1).

**TABLE 1.**
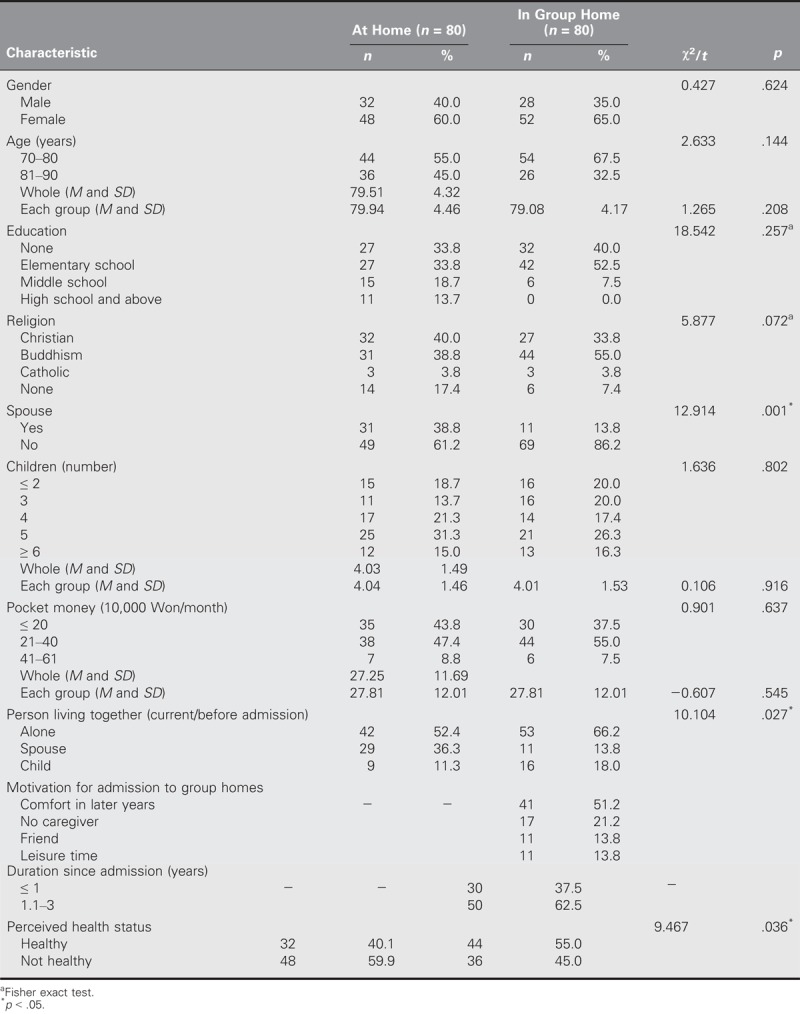
General Characteristics of Study Participants (*N* = 160)

### Differences of Health Promoting Behaviors, Depression, and Life Satisfaction Between the Two Groups

Participation in health promoting behaviors was significantly higher in the group-home group than in the at-home group (*t* = −9.035, *p* < .001), level of depression was significantly lower in the group-home group than in the at-home group (*t* = 20.861, *p* < .001), and life satisfaction was significantly higher in the group-home group than in the at-home group (*t* = −12.153, *p* < .001; Table 2).

**TABLE 2.**
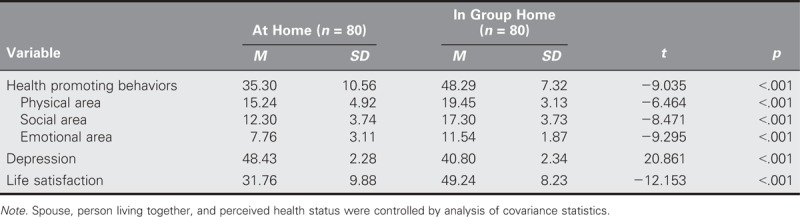
Differences in Health Promoting Behaviors, Depression, and Life Satisfaction Between Older Adults in Group Homes and at Home (*N* = 160)

### Intergroup Comparisons of Differences in Health Promoting Behaviors, Depression, and Life Satisfaction According to General Characteristics

With regard to the health promoting behaviors of the at-home group, significant differences were identified in terms of gender (*t* = 2.678, *p* = .009), educational level (*t* = 7.723, *p* < .001), having a spouse (*t* = 5.620, *p* < .001), living with another person (*t* = 3.044, *p* = .034), and perceived health status (*t* = 6.480, *p* < .001). With regard to the health promoting behaviors of the group-home group, significant differences were identified in terms of age (*t* = 5.154, *p* < .001), educational level (*t* = 11.398, *p* < .001), religion (*t* = 2.765, *p* = .048), having a spouse (*t* = −3.461, *p* = .001), and perceived health status (*t* = 6.055, *p* < .001; Table 3).

**TABLE 3.**
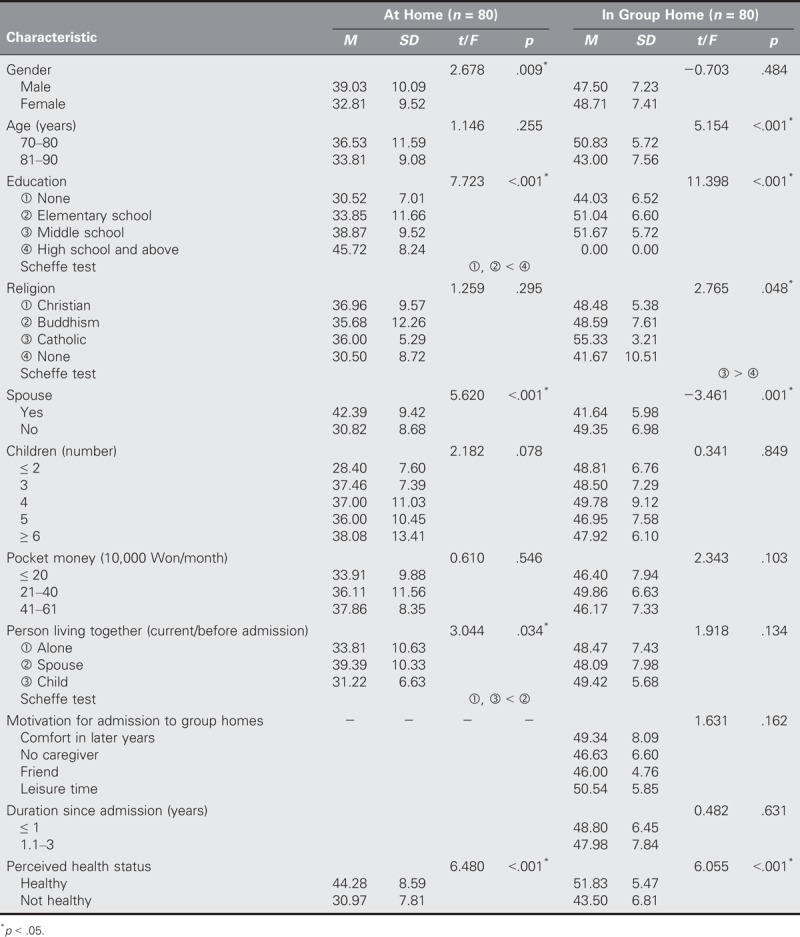
Comparisons of Differences in Health Promoting Behavior by General Characteristics of Older Adults Between Older Adults in Group Homes and at Home (*N* = 160)

With regard to depression, significant differences were found in terms of living with another person (*t* = 0.251, *p* = .043) for the at-home group and in terms of gender (*t* = −1.978, *p* = .047) and having a spouse (*t* = 2.308, *p* = .024) for the group-home group (Table 4). Finally, with regard to life satisfaction, significant differences were found in terms of gender (*t* = 4.827, *p* < .001), educational level (*t* = 16.573, *p* < .001), having a spouse (*t* = 6.701, *p* < .001), pocket money (*t* = 3.327, *p* = .041), living with another person (*t* = 7.437, *p* < .001), and perceived health status (*t* = 12.386, *p* < .001) for the at-home group and in terms of age (*t* = 4.301, *p* < .001), having a spouse (t = −3.432, *p* = .001), and perceived health status (*t* = 6.197, *p* < .001) for the group-home group (Table 5).

**TABLE 4.**
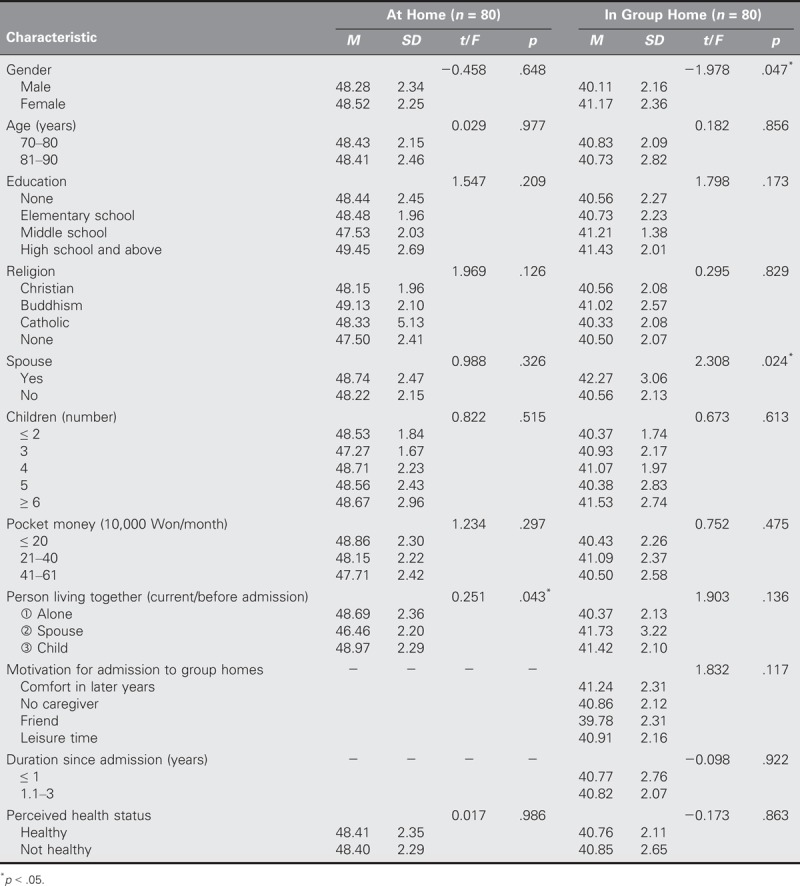
Comparisons of Differences in Depression by General Characteristics Between Older Adults in Group Homes and at Home (*N* = 160)

**TABLE 5.**
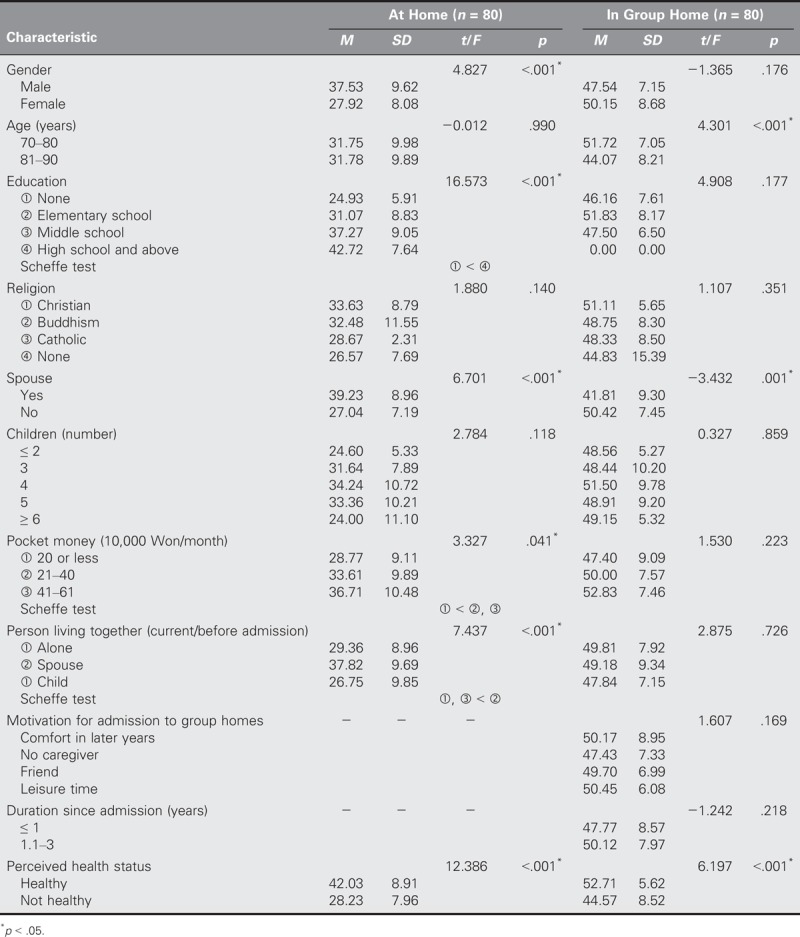
Comparisons of Differences in Life Satisfaction by General Characteristics Between Older Adults in Group Homes and at Home (*N* = 160)

## Discussion

The comparison in this study of older adults living at home with older adults living in group homes in rural areas showed that the level of health promoting behaviors among the latter was higher than that of the former. In all of the subcategories of health promoting behaviors, including the physical, social, and emotional dimensions, the level of health promoting behaviors was higher in the group-home group than in the at-home group. Considering that personal health is an important factor affecting friendship relationships (Chang & Park, 2012; Schulz, Boerner, Klinger, & Rosen, 2015; Shearer, Fleury, Ward, & O’Brien, 2012), older adults who live at home in rural areas of South Korea are more limited and disadvantaged than their peers who live in group homes in terms of the frequency and degree of interpersonal interactions (Ko, 2016; Sok & Kim, 2008). Therefore, older adults living in group homes are in a much better position to develop a base of support through interactions with fellow residents and to improve self-esteem and improve their health promoting behaviors through information sharing. Comparing the health promoting behaviors of the two groups based on general characteristics showed that health promoting behaviors were better at lower ages, at higher levels of education, and at higher self-perceived levels of health. These results echoed those of H. S. Shin (2007), which targeted older adults who used senior citizen or welfare centers. These findings support that more health-related information should be provided to older adults who are less educated and who have health problems.

The comparison in this study of depression levels in the two groups found a depression level of 48.43 in the at-home group, which was significantly higher than that of the group-home group (40.80). On the basis of the criteria of the depression scale, the group-home group had “moderate depression” and the at-home group had “severe depression.” In Mirotznik and Kamp (2000), the stress of environmental change appeared as a negative emotion in older adults who had been admitted to a facility. In Hong (2008), the depression level of older adults living in facilities was higher than that of the older adults living at home because the children notified their parents of their decision to sending the latter to a nursing home after a discussion. The lower level of depression in the group-home group in this study may be due to their living in an environment similar to where they used to live and to their choosing to move into a group home. Living in group homes is likely to give residents more opportunities to obtain support and positive reinforcement from interactions with fellow residents who are in similar positions in activities such as self-help groups for patients with incurable diseases. This may help explain why the group-home group self-reported lower levels of depression than the at-home group. In terms of personal characteristics, depression was higher in female than male participants. According to data from the Health Insurance Review and Assessment Service (2013), approximately 70% of patients with depression aged 60 years or older are female, which is more than twice the percentage of their male peers. In addition, in terms of spousal status, more participants with a spouse reported depression than those who did not. Most in the group-home group with a spouse were in an unpleasant situation, as the spouse had been admitted to a hospital because of a geriatric disease or under situations where they lived separately because of marital conflict. These factors may explain their higher level of depression.

Furthermore, the group-home group reported a higher satisfaction with life than the at-home group. In another study that compared the life satisfaction of older adults living in a senior citizen welfare town with that of older adults living at home, although no difference was found in overall life satisfaction, the town-dwellers scored higher in several subdimensions, including emotional support and experience satisfaction, which supports the findings of this study (H. S. Kim, 2016; S. H. Lee, 2010). The reason for this seems to be that, unlike the older adults living at home, those living in group homes have ready access to assistance in emergency situations. On the other hand, Custers et al. (2010) found that older adults who had been admitted to a facility had unpleasant life experiences and had lower self-reported life satisfaction, which differs from this study. The reason for this seems to be that group homes, unlike general nursing home facilities, strive to provide familiar or similar environments for residents, thus providing greater life satisfaction. In addition, considering previous studies that identified interpersonal relationships with fellow residents as an important factor for improving life satisfaction at the facility (Chang & Park, 2012; Cho et al., 2017; Koppitz et al., 2017), older adults living in group homes in rural South Korea are more likely than their at-home peers to receive positive support and build intimacy through interpersonal communications and thus report better life satisfaction. This study further found that life satisfaction in the group-home group decreased with age, which is consistent with expectations. In Son (2006), better perceived physical health status was shown to be positively associated with life satisfaction, supporting the results of this study. For older adults with a spouse living at home, their life satisfaction was higher than that of older adults without a spouse at home, which was similar to the finding of S. H. Shin and Sok (2012) that life satisfaction was higher when the quality of family relationships was better. On the other hand, in the case of the older adults living in group homes, those without a spouse, who accounted for more than half of the older adult residents, reported higher life satisfaction than those with a spouse. As for those in the group-home group who had a spouse, the spouse was often currently hospitalized because of a disease or lived separately because of marital conflict, which may explain this finding.

In the impact on the at-home group of having a spouse on life satisfaction, health promotion, and depression, it was more positive in terms of health promoting behaviors and life satisfaction than in the their at-home peers without a spouse. This finding echoed that of previous studies (Custers et al., 2010; S. M. Lee, 2012; S. H. Shin & Sok, 2012). On the other hand, the older adults with a spouse in group homes had lower health promoting behaviors and life satisfaction than their group-home peers without a spouse. This finding also echoed previous studies (Cho et al., 2017; Vogelsang, 2016). In terms of depression, older adults with a spouse in group homes reported more depression than their peers without a spouse in group homes. These findings are explained by the fact that older adults living at home in rural South Korea receive positive impacts from their spouses and that older adults living in group homes in rural South Korea are influenced positively by relationships with fellow friends rather than with their spouse. In addition, positive findings on life satisfaction, health promotion, and depression in older adults living in group homes in rural South Korea may be the result of the structural modification by service interventions of the lifestyles of people living in group homes.

### Limitations

This study is limited in terms of the representativeness and generalizability of the sample population. The samples used in this study were recruited only from K rural areas in South Korea, which limits the characteristics of the resultant data. However, the essential aim of this study was not to generate generalizable results but rather to provide information on which to frame future research.

### Conclusions

The findings from this study may be used as basic data to establish nursing interventions to improve the prospects for healthy aging and life satisfaction by accurately identifying the needs and health problems of older adults living in rural areas, categorized by residence type. Especially, the incidence of depression was lower among older adults living in group homes than among older adults living at home. However, both groups self-reported as having moderate–severe depression. On the basis of this, a nursing intervention plan needs to be prepared to reduce the depression of older adults in the rural areas. Systematic and intensive management is required for older adults as well as adults throughout the human lifecycle. Health policies must focus on the health issues of older adults, especially those in rural South Korea. In terms of policy implications, a government agency should formulate health policies that encourage group-home facilities to be built throughout rural areas in South Korea.

In conclusion, health promotion behaviors and life satisfaction were higher and the incidence of depression was lower among older adults living in group homes than among older adults living at home in rural South Korea. Therefore, more group home facilities should be built in rural areas where the older adult population is rapidly increasing. Further studies are necessary to characterize the lives of the older adults living in group homes in rural areas by comparing their life before admission to the group homes and after their admission. Studies to investigate the impact of having a spouse on older adults living at home and the impact of relationships with fellow friends on older adults living in group homes in rural South Korea are also needed. In this study, we assumed that interactions and greater social support are two key factors that explain why people living in group homes rated all three variables better. These assumptions should be further studied in the future. Furthermore, in-depth, qualitative studies are required to understand and analyze the inner world and life experiences of older adults living at home and in group homes in rural areas.
